# Fecal Microbiota and Volatile Metabolome Pattern Alterations Precede Late-Onset Meningitis in Preterm Neonates

**DOI:** 10.1093/infdis/jiae265

**Published:** 2024-05-23

**Authors:** Nina M Frerichs, Nancy Deianova, Sofia el Manouni el Hassani, Animesh Acharjee, Mohammed Nabil Quraishi, Willem P de Boode, Veerle Cossey, Christian V Hulzebos, Anton H van Kaam, Boris W Kramer, Esther d’Haens, Wouter J de Jonge, Daniel C Vijlbrief, Mirjam M van Weissenbruch, Emma Daulton, Alfian N Wicaksono, James A Covington, Marc A Benninga, Nanne K H de Boer, Johannes B van Goudoever, Hendrik J Niemarkt, Tim G J de Meij

**Affiliations:** Department of Pediatric Gastroenterology, Emma Children's Hospital, Amsterdam University Medical Center, Amsterdam, The Netherlands; Amsterdam Gastroenterology Endocrinology Metabolism Research Institute, Amsterdam University Medical Center, Amsterdam, The Netherlands; Amsterdam Reproduction and Development Research Institute, Amsterdam University Medical Center, University of Amsterdam, Amsterdam, The Netherlands; Department of Pediatric Gastroenterology, Emma Children's Hospital, Amsterdam University Medical Center, Amsterdam, The Netherlands; Amsterdam Gastroenterology Endocrinology Metabolism Research Institute, Amsterdam University Medical Center, Amsterdam, The Netherlands; Amsterdam Reproduction and Development Research Institute, Amsterdam University Medical Center, University of Amsterdam, Amsterdam, The Netherlands; Department of Pediatric Gastroenterology, Emma Children's Hospital, Amsterdam University Medical Center, Amsterdam, The Netherlands; Amsterdam Gastroenterology Endocrinology Metabolism Research Institute, Amsterdam University Medical Center, Amsterdam, The Netherlands; Amsterdam Reproduction and Development Research Institute, Amsterdam University Medical Center, University of Amsterdam, Amsterdam, The Netherlands; College of Medical and Dental Sciences, Institute of Cancer and Genomic Sciences, University of Birmingham, Birmingham, United Kingdom; Institute of Translational Medicine, University Hospitals Birmingham NHS Foundation Trust, Birmingham, United Kingdom; MRC Health Data Research UK (HDR UK), Birmingham, United Kingdom; Institute of Cancer and Genomic Sciences, University of Birmingham, Birmingham, United Kingdom; Neonatal Intensive Care Unit, Radboud University Medical Center, Radboud Institute for Health Sciences, Amalia Children's Hospital, Nijmegen, The Netherlands; Neonatal Intensive Care Unit, University Hospitals Leuven, Leuven, Belgium; Neonatal Intensive Care Unit, Beatrix Children's Hospital, University Medical Center Groningen, Groningen, The Netherlands; Amsterdam Reproduction and Development Research Institute, Amsterdam University Medical Center, University of Amsterdam, Amsterdam, The Netherlands; Department of Neonatology, Emma Children's Hospital, Amsterdam University Medical Center, Amsterdam, The Netherlands; Department of Pediatrics, Maastricht University Medical Center, Maastricht, The Netherlands; Neonatal Intensive Care Unit, Amalia Children’s Center, Isala, Zwolle, The Netherlands; Tytgat Institute for Liver and Intestinal Research, Amsterdam University Medical Center, Amsterdam, The Netherlands; Department of Surgery, University of Bonn, Bonn, Germany; Neonatal Intensive Care Unit, Wilhelmina Children's Hospital, University Medical Center Utrecht, Utrecht, The Netherlands; Department of Neonatology, Emma Children's Hospital, Amsterdam University Medical Center, Amsterdam, The Netherlands; School of Engineering, University of Warwick, Coventry, United Kingdom; School of Engineering, University of Warwick, Coventry, United Kingdom; School of Engineering, University of Warwick, Coventry, United Kingdom; Department of Pediatric Gastroenterology, Emma Children's Hospital, Amsterdam University Medical Center, Amsterdam, The Netherlands; Amsterdam Gastroenterology Endocrinology Metabolism Research Institute, Amsterdam University Medical Center, Amsterdam, The Netherlands; Department of Gastroenterology and Hepatology, Amsterdam University Medical Center, Vrije Universiteit, Amsterdam, The Netherlands; Department of Pediatric Gastroenterology, Emma Children's Hospital, Amsterdam University Medical Center, Amsterdam, The Netherlands; Amsterdam Gastroenterology Endocrinology Metabolism Research Institute, Amsterdam University Medical Center, Amsterdam, The Netherlands; Neonatal Intensive Care Unit, Máxima Medical Center, Veldhoven, The Netherlands; Department of Pediatric Gastroenterology, Emma Children's Hospital, Amsterdam University Medical Center, Amsterdam, The Netherlands; Amsterdam Gastroenterology Endocrinology Metabolism Research Institute, Amsterdam University Medical Center, Amsterdam, The Netherlands

**Keywords:** late-onset meningitis, preterm neonates, volatile organic compounds, microbiota analysis, fecal biomarker

## Abstract

**Background:**

The fecal microbiota and metabolome are hypothesized to be altered before late-onset neonatal meningitis (LOM), analogous to late-onset sepsis (LOS). The present study aimed to identify fecal microbiota composition and volatile metabolomics preceding LOM.

**Methods:**

Cases and gestational age-matched controls were selected from a prospective, longitudinal preterm cohort study (born <30 weeks’ gestation) at 9 neonatal intensive care units. The microbial composition (16S rRNA sequencing) and volatile metabolome (gas chromatography-ion mobility spectrometry [GC-IMS] and GC-time-of-flight-mass spectrometry [GC-TOF-MS]) were analyzed in fecal samples 1–10 days pre-LOM.

**Results:**

Of 1397 included infants, 21 were diagnosed with LOM (1.5%), and 19 with concomitant LOS (90%). Random forest classification and MaAsLin2 analysis found similar microbiota features contribute to the discrimination of fecal pre-LOM samples versus controls. A random forest model based on 6 microbiota features accurately predicted LOM 1–3 days before diagnosis with an area under the curve (AUC) of 0.88 (n = 147). Pattern recognition analysis by GC-IMS revealed an AUC of 0.70–0.76 (*P* < .05) in the 3 days pre-LOM (n = 92). No single discriminative metabolites were identified by GC-TOF-MS (n = 66).

**Conclusions:**

Infants with LOM could be accurately discriminated from controls based on preclinical microbiota composition, while alterations in the volatile metabolome were moderately associated with preclinical LOM.

## Teaser

The fecal microbiota and volatile metabolome are altered in preterm infants prior to late-onset meningitis. A prediction model based on 6 bacterial genera and species could accurately predict late-onset meningitis 1–3 days before clinical onset with an AUC of 0.88.

**(See the Editorial Commentary by Whiteside and John on pages 1349–52.)**

Late-onset neonatal meningitis (LOM), a potentially lethal central nervous system (CNS) infection, is a complication of prematurity with a mortality rate of around 25% [1]. The incidence of approximately 2 per 1000 among very preterm infants is likely an underestimate due to diagnostic difficulties of LOM [2, 3]. Among survivors, long-term neurological sequelae are estimated to occur in 24%–58% of cases, largely depending on the causative pathogen [3–5].

Although cerebrospinal fluid (CSF) culture is considered the gold standard for LOM diagnosis, lumbar puncture sampling can be challenging and contraindicated, for example, in hemodynamically unstable or thrombocytopenic patients [6]. Consequently, life-saving, empiric high-dose broad-spectrum antibiotic therapy is administered based on clinical suspicion, compromising interpretation of future CSF culturing [3, 7]. Even with timely CSF collection, culture results take 48–72 hours to interpret, and clinicians base the initial diagnosis on biochemical parameters such as CSF white blood cell (WBC) count, and protein and glucose concentrations. However, interpretation of CSF parameters is often complicated in preterm infants by traumatic lumbar punctures, presence of intraventricular hemorrhage, or earlier exposure to antibiotics [8]. To prevent potential adverse outcome due to delay in diagnosis and initiation of treatment, exploring new rapid, preferably noninvasive, diagnostic biomarkers based on pathophysiological insights is imperative [9].

Prior to the clinical onset of other acute diseases in preterm neonates, including necrotizing enterocolitis (NEC) and late-onset sepsis (LOS), the gut microbiota composition and the associated volatile metabolome exhibit alterations [10–18]. Part of the LOS cases are thought to originate from the gut, supported by findings of genetic similarities between blood-cultured pathogens and gut-residing bacteria before diagnosis [17, 19]. Delay in microbiota maturation, as commonly observed in preterm neonates, is hypothesized to favor the overgrowth of pathogenic bacteria, contributing to bacterial translocation across the immature epithelial and endothelial layers into the bloodstream [20–23]. Furthermore, age-dependent incomplete polarization of the choroid plexus epithelium in neonates may encourage bacterial translocation over the blood-brain barrier, eventually leading to LOM [22].

Because co-occurrence of LOS is observed in two-thirds of infants with LOM [3], we hypothesize that fecal microbiota and the volatile metabolome are altered before LOM diagnosis. Analogous to LOS, we hypothesize that LOM-causing pathogens reside and potentially overgrow in the gut before translocation into the CSF via the bloodstream. The aim of this study was to explore the volatile metabolome and longitudinal composition of the gut microbiota preceding LOM in preterm infants to investigate its potential contribution to LOM pathophysiology and diagnostic value. The volatile metabolome was assessed using 2 complementary techniques, gas chromatography-ion mobility spectrometry (GC-IMS), a pattern recognition-based technique, and gas chromatography-time of flight-mass spectrometry (GC-TOF-MS), for unique metabolite identification.

## METHODS

### Subjects

This study is part of a prospective multicenter cohort focused on infants born before 30 weeks of gestation in 9 neonatal intensive care units in the Netherlands and Belgium. The primary objective of this ongoing cohort study is discovering novel noninvasive biomarkers for LOS and NEC [10–14]. For this study, daily fecal samples and clinical data were collected in the first 28 days of life. Exclusion criteria of this cohort study are major congenital gastrointestinal diseases. The study was approved by the local medical ethical review boards (protocol 2014.386, amendment A2016.363) and written informed consent was obtained from parents or legal guardians.

For the current case-control study, all infants born between October 2014 and December 2018 with diagnosed LOM were included. Exclusion criteria were early onset sepsis, NEC, or spontaneous intestinal perforation. In control infants, diagnosis of LOS during the 28-day study period was an additional exclusion criterion.

The definition of LOM (onset ≥72 hours after birth) was adapted from the “CDC/NHSN Surveillance Definitions for Specific Types of Infections” [24]. At least criteria 1, 2 (a, b, or c), and 3 needed to be fulfilled: (1) clinical signs of generalized infection (temperature instability, apnea, bradycardia, irritability/lethargy); (2a) a positive CSF culture or (2b) a positive blood culture or (2c) C-reactive protein levels >10 mg/L; and (3) CSF WBC count > 30/µL. Additionally, systemic antibiotic treatment needed to be initiated with the intention to treat for LOM for ≥7 consecutive days. All cases were matched to controls based on center of birth, gestational age (±2 days), birth weight (±150 grams), and exact postnatal age. LOS was defined according to the Vermont-Oxford criteria: (1) clinical signs of generalized infection, (2) a positive blood culture ≥72 hours after birth, and (3) initiation of antibiotic treatment with the intention to treat for ≥5 consecutive days [25].

**Figure 1.**
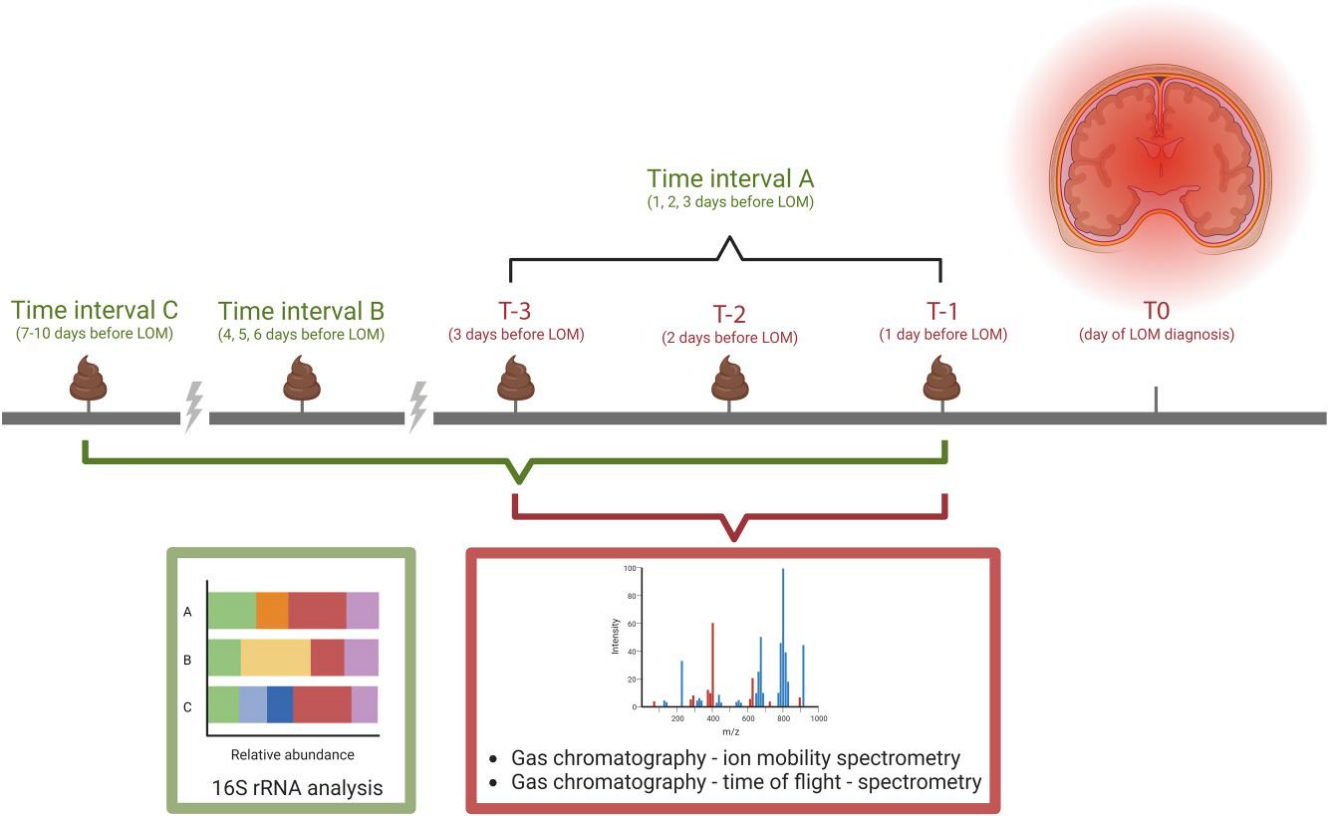
Selected time points for microbiota and metabolomics analysis. Fecal samples were analyzed in 3 groups based on the time interval between sampling and onset of late-onset meningitis (LOM) (T0). Three time intervals (TI) were defined for microbiota analysis on samples from up to 10 days before clinical onset of LOM and controls: TI-A (1 to 3 days before onset), TI-B (4 to 6 days before onset), and TI-C (7 to 10 days before onset). Volatile metabolomics was performed on 3 days before disease onset, where T-3, T-2, and T-1 represented 3, 2, and 1 days prior to LOM onset, respectively. Figure created with Biorender.com.

**Figure 2.**
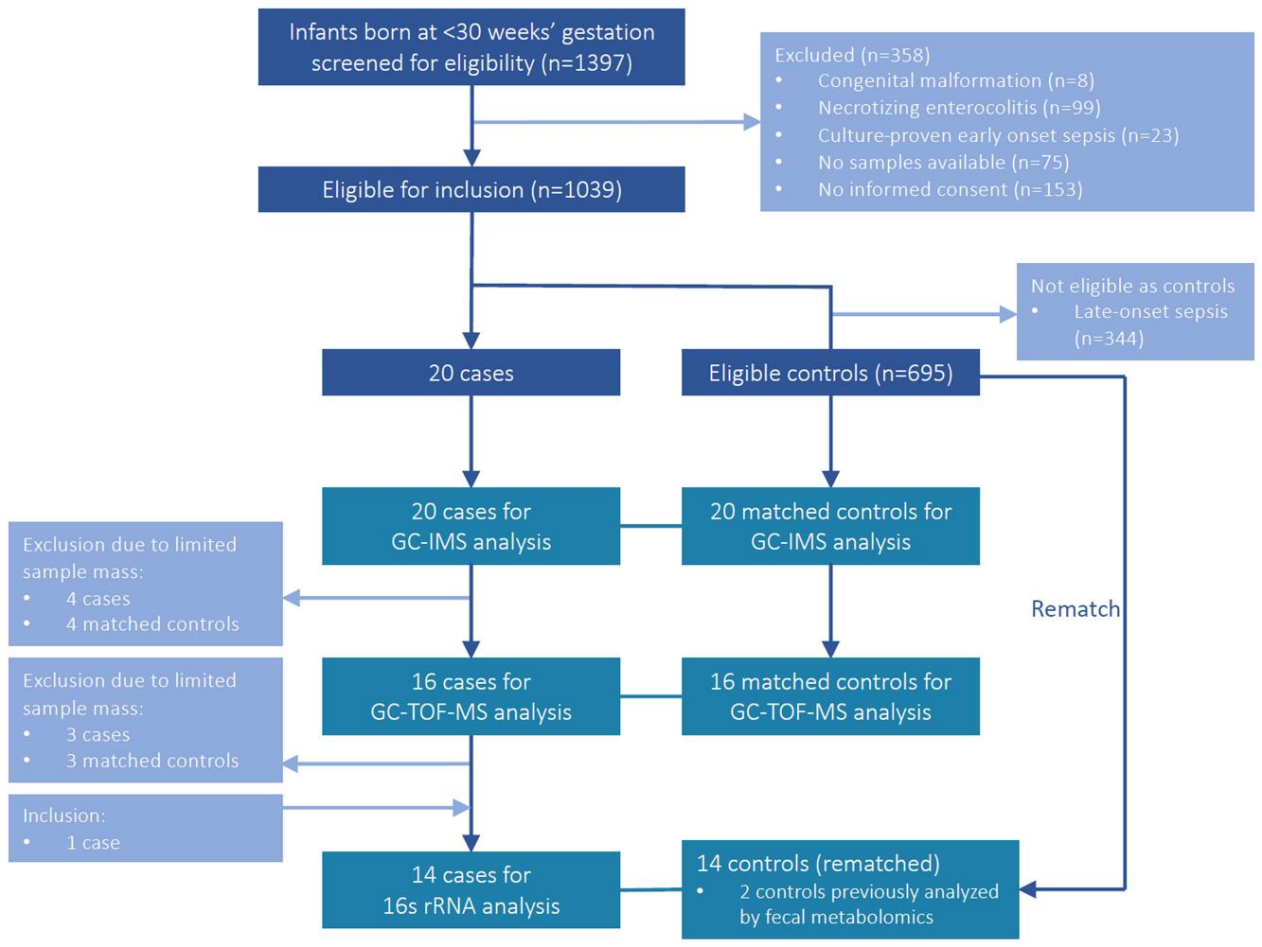
Flowchart of the inclusion and case-control matching process. In total, premeningitis samples of 21 children who developed late-onset meningitis were analyzed, while the fecal samples of 32 control infants were analyzed. Cases were first analyzed by GC-IMS (20 cases matched to 20 controls). Four match pairs were excluded from subsequent GC-TOF-MS analysis due to limited sample mass, resulting in 16 cases and 16 matched controls. 16S rRNA sequencing was conducted at a later time point. Three match pairs were excluded from this analysis due to limited sample mass, and 1 additional case was added, resulting in 14 cases with enough fecal matter available for 16S rRNA analysis. These cases were rematched to controls with sufficient sample mass, resulting in 14 matched controls. Of these 14 controls, 2 were previously analyzed by volatile metabolomics. Abbreviations: 16S rRNA,16S ribosomal ribonucleic acid; GC-IMS, gas chromatography-ion mobility spectrometry; GC-TOF-MS, gas chromatography-time of flight-mass spectrometry. Figure created with Biorender.com.

Cases and matched controls were primarily selected for volatile metabolome analysis with samples available from 1–3 days before clinical onset of LOM. This timeframe aligns with previous studies on NEC and LOS, indicating that the highest discriminating potential of volatile organic compounds (VOC) is 1–3 days before clinical onset [11, 12]. Microbiota analysis included samples up to 10 days before diagnosis to explore longitudinal profiles (Figure 1). Because several cases and controls did not have enough fecal matter present after the volatile metabolome analyses, only the cases with sufficient material left were included for microbiota analysis, and matched to new controls. This resulted in a larger number of controls, implying a matching approach diverging from the exact 1:1 ratio; however, for all 3 analytical techniques separate the cases are matched 1:1 to controls (Figure 2). One LOM case with clinical data collected after volatile metabolome analysis was included in microbiota analysis (Figure 2).

### Sample and Data Collection

Fecal samples were collected once daily, from birth up to 28 days, in a polystyrene container (Stuhlgefäß 10 mL), and stored at −20°C within an hour. Sample collection ceased in cases of transfer to a referral hospital or death. Only samples with weight >25 mg were included. As previously described, clinical data, including health events, medication use, and feeding type, were collected daily [10–14]. A feeding type with significant amount of formula milk was defined as >25% of the average daily enteral intake consisting of formula milk. Probiotics were not routinely administered in any participating center.

Before volatile metabolomics analysis, the fecal samples were transported from the original collection tube into sterile glass vials (20 mL headspace vial; Thames Restek) to the School of Engineering at the University of Warwick (Coventry, United Kingdom). Upon arrival, fecal samples were stored at −20°C until further handling. Samples were analyzed in June 2019. Before performing 16S rRNA sequencing, fecal samples were shipped in their original collection tube to the University of Birmingham (Birmingham, United Kingdom). These samples were also stored at −20°C until further handling. Samples were analyzed in December 2019. The samples were always shipped on dry ice (−78.5°C).

### Fecal Microbiota 16S rRNA Profiling

Extracted paired DNA was used for 16S rRNA gene amplification and sequencing, using the Qiagen AllPrep DNA/RNA Mini Kit, strictly following the Earth Microbiome Project protocol [26, 27]. Using primers targeting the 16S rRNA V4 region (515F-806R) in a 1-step, single-indexed polymerase chain reaction (PCR) approach, the 16S rRNA genes were amplified in duplicates. This was done in batches, using the appropriate negative controls. Subsequently, paired-end sequencing (2 × 250 bp) was performed on an Illumina MiSeq platform and processed in the pipeline Quantitative Insights Into Microbial Ecology 2 (QIIME 2) [28]. Taxonomy was assigned against the Silva 132 99% operating taxonomic units (OTUs) database [29].

### Fecal Volatile Metabolome Analysis

Two different techniques were used for fecal volatile metabolome analysis. First, all samples were analyzed by GC-IMS to measure and compare VOC patterns of LOM versus controls. Next, GC-TOF-MS was used to identify unique metabolites in the headspace of fecal samples by a nontargeted approach through measuring mass-to-charge ratio of volatile metabolites. The workflow and settings for both techniques are described in Supplementary Material 1 and 2.

### Statistical and Machine Learning Analysis

For statistical analyses of demographic and clinical data, Statistical Package for the Social Sciences (SPSS) version 26 (IBM) was used. When considered appropriate based on the discrete versus continuous (either Gaussian or non-Gaussian) character of data, χ^2^ test, independent Student *t* test or Mann-Whitney *U* test were applied to calculate *P* values. A *P* value <.05 was considered significant for all methods used.

For microbiota analysis, the samples were divided into 3 comparison groups based on the time of sampling prior to onset of LOM (Figure 1) as follows:

- Time interval A (TI-A) contains samples from 1, 2, and 3 days before diagnosis.
- Time interval B (TI-B) contains samples from 4, 5, and 6 days before diagnosis.
- Time interval C (TI-C) contains samples from 7, 8, 9, and 10 days before diagnosis.

The volatile metabolome analysis was performed on samples collected 1, 2, and 3 days before diagnosis (TI-A) (Figure 1).

The preprocessing of the microbiota data is described in Supplementary Material 3*A*. Principal coordinates analysis (PCoA), PERMANOVA, random forest (RF), and MaAsLin2 analysis are described in Supplementary Material 3*B–E*. Volatile metabolomics data were preprocessed as described in Supplementary Material 3*F*. Subsequently, class prediction by RF was performed on GC-IMS data, and the GC-TOF-MS data were analyzed by Student *t* test for unequal variances. The class prediction method is described in Supplementary Material 3*G*.

## RESULTS

### Baseline Characteristics

In total, 1397 preterm neonates were included in the study of whom 21 were diagnosed with LOM in the first 28 days of life (1.5%) (Figure 2). For GC-IMS analysis, 41 and 51 samples, and for GC-TOF-MS, 32 and 34 samples were analyzed from cases and controls, respectively, ranging from 1 to 3 days before diagnosis. For microbiota analysis, 69 and 78 case and control samples were analyzed, respectively, ranging from 1 to 10 days before diagnosis. Supplementary Table 1 displays the total number of samples per case per technique.

Subjects’ baseline characteristics are depicted in Table 1. Nineteen cases (90%) had an associated blood-culture–proven LOS episode within 48 hours before, after, or during LOM development, with overlapping LOS- and LOM-causing pathogens in 6 neonates. Notably, 14 out of 21 infants had received antibiotic treatment <48 hours before lumbar puncture, and thus before CSF culture was obtained. No infants had indwelling cranial devices before LOM. Detailed clinical information on the LOM infants is included in Supplementary Table 1.

**Table 1.** Baseline Clinical and Demographic Characteristics per Analytical Technique

Characteristics	Total Late-Onset Meningitis Cases (n = 21)	Fecal Volatile Metabolome			Fecal Microbiota		
		Late-Onset Meningitis (n = 20)	Controls (n = 20)	*P* Value Volatile Metabolome	Late-Onset Meningitis (n = 14)	Controls (n = 14)	*P* Value Microbiota
Gestational age, weeks + days, median (Q1–Q3)	27 + 4 (25 + 4–28 + 6)	27 + 5 (25 + 6–28 + 6)	27 + 3 (25 + 6–29 + 0)	.807	27 + 5 (25 + 2–28 + 6)	27 + 3 (25 + 5–29 + 0)	.765
Delivery mode, CD, n (%)	12 (57)	12 (60)	5 (25)	.025*	7 (50)	4 (29)	.246
Sex, female, n (%)	10 (48)	10 (50)	11 (55)	.752	6 (43)	7 (50)	.705
Singleton, n (%)	14 (67)	14 (70)	12 (60)	.507	8 (57)	7 (50)	.705
Birth weight, gram, median (Q1–Q3)	1010 (745–1145)	1030 (781–1153)	1050 (800–1168)	.935	1030 (748–1179)	1003 (839–1215)	.963
Meningitis, day of life, median (Q1–Q3)	13 (9–19)	13 (8–19)	NA	NA	14 (10–20)	NA	NA
Associated late-onset sepsis, n (%)	19 (91)	18 (90)	NA	NA	12 (86)	NA	NA
Formula milk administered before T0, n (%)	7 (33)	7 (35)	10 (50)	.491	5 (36)	4 (29)	.528
Antibiotic duration before T0, d, median (Q1-Q3)	4 (3–7)	4 (3–7)	5 (3–6)	.934	4 (3–7)	3 (3–5)	.688

Missing values are considered in the percentage calculation.

Abbreviations: CD, cesarean delivery; Q1*–*Q3, quartile 1*–*quartile 3; T0, day of meningitis diagnosis.

^*^ *P* value < .05.

### Microbiota Analysis

The 16S rRNA data were first plotted at phylum level to visually explore results. Supplementary Figure 1 shows the relative abundance of the phyla in cases and controls for 1 to 10 days before diagnosis of LOM or the corresponding postnatal days in controls. The microbiota in LOM infants seems to be characterized by increased relative abundance of Proteobacteria and decreased Firmicutes and Bacteriodetes, compared to controls. Diversity parameters were comparable between cases and controls (Supplementary Figure 2).

A PCoA score plot was performed using Bray-Curtis dissimilarity distance to demonstrate variations across samples (LOM vs controls) and time intervals. PERMANOVA analysis found significant differences (*P* value = .002) between the control vs disease samples when all TIs were pooled together, whereas separately only TI-C is significant (*P* value < .022). PCoA analysis of the 3 TIs separately and the pooled TIs together are shown in Supplementary Figure 3.

Then, RF classification and MaAsLin2 analysis were performed to rank the OTU abundance based on variable importance and *P* values, respectively. RF analysis selected 22 OTUs as contributing to the discrimination of LOM versus control samples across different TIs (Supplementary Table 2). The top 7 features (*P* value < .1) as analyzed by MaAslin2 are depicted in Supplementary Table 3. Six top features were overlapping between methods (Table 2). *Bacteroides* genus and the Yersiniaceae family were important in TI-A. In TI-B, *Leuconostoc mesenteroides* species and the Yersiniaceae family were selected, whereas the genera *Corynebacterium* and *Staphylococcus* were important features in TI-C. Supplementary Figure 4 displays the up- and downregulation of the selected features. Supplementary Figure 5 depicts the OTUs that are the most discriminative between LOM and controls as calculated by the RF model for the 3 separate TIs. The complete MaAsLin2 outcomes for the 3 separate TIs, including coefficients, standard error, *P* values, and Q values, are shown in Supplementary Tables 4–6.

**Table 2.** Families, Genera, and Species Ranked as Highest Discriminating Features Between Late-Onset Meningitis Samples and Matched Controls by Random Forest Analysis (Variable Importance) and MaAsLin2 Analysis (*P* Value < .1) on the Separate Time Intervals

Phylogenic Level	Microbiota Feature	TI-A	TI-B	TI-C
Family	Yersiniaceae	X	X	…
Genus	*Bacteroides*	X	…	…
	*Staphylococcus*	…	…	X
	*Corynebacterium*	…	…	X
Species	*Leuconostoc mesenteroides*	…	X	…

Abbreviation: TI, time interval.

We additionally used an RF class prediction to identify a set of specific OTUs that are able to discriminate LOM from control samples among the different TIs, as summarized in Table 3. At all TIs, samples from LOM infants could be distinguished from control samples by the RF classification model, with the highest accuracy in TI-A with an area under the curve (AUC) of 0.88 (95% confidence interval [CI], .71–.98).

**Table 3.** Families, Genera, and Species Contributing to the Random Forest Class Prediction Model on the Separate Time Intervals and Upward/Downward Trend per Time Interval

Phylogenic Level	Family/Genus/Species	TI-A	TI-B	TI-C
Family	Yersiniaceae	↑	↑	…
	Comamonadaceae	…	↓	↓
Genus	*Bacteroides*	↓	…	…
	*Enterococcus*	↑	…	…
	*Escherichia/Shigella*	↑	…	↑
	*Veillonella*	↑	↓	…
	*Ralstonia*	…	↓	↓
	*Staphylococcus*	…	↓	↓
Species	*Afipia* uncultured species	…	…	↓
	*Leuconostoc mesenteroides*	↑	↑	…
	*Streptococcus salivarius*	…	↓	…
AUC (95% CI)		0.88 (.71–.98)	0.74 (.57–.90)	0.81 (.58–.98)
Sensitivity		1.0	1.0	0.81
Specificity		0.5	0.45	0.66
OOB error rate, %		25.5	33.3	26.7

Abbreviations: AUC, area under the curve; CI, confidence interval; OOB, out of bag error; TI, time interval.

Next, the top 5 abundant OTUs (ie, species, genus, family) in every infant with LOM was identified (Supplementary Table 7). A positive CSF culture was found in 8 of the 14 infants with LOM. In 6 cases (75%), 1 of the top 5 most prevalent OTUs in the gut microbiome represented the OTU that most closely matched the causal agent found in the CSF. For example, the genus *Escherichia-Shigella* was found as a top 5 abundant genus in 2 infants with *Escherichia coli* LOM.

### Fecal Volatile Metabolome Analysis by GC-IMS

Volatile metabolome patterns of fecal samples derived from infants developing LOM were significantly different compared to controls as analyzed by RF analysis using 20 features, with AUC ranging from 0.70 to 0.76 (Table 4). Supplementary Table 8 shows the effect of using 50 and 100 features.

**Table 4.** Performance Characteristics of Gas-Chromatography-Ion Mobility Spectrometry as Analyzed by Random Forest With 20 Features

Time Point, d	Control Samples, n	Case Samples, n	*P* Value	AUC (95% CI)	Sensitivity (95% CI)	Specificity (95% CI)	PPV	NPV
T-1	15	18	.01	0.74 (.57–.91)	0.60 (.32–.84)	0.83 (.59–.96)	0.75	0.71
T-2	13	19	.03	0.70 (.51–.90)	0.38 (.14–.68)	1.00 (.82–1.00)	1.00	0.70
T-3	13	14	.01	0.76 (.57–.94)	0.92 (.64–1.00)	0.57 (.29–.82)	0.67	0.89

Abbreviations: CI, confidence interval; NPV, negative predictive value; PPV, positive predictive value; T-1, T-2, T-3, the day before, 2 days before, and 3 days before clinical diagnosis of late-onset meningitis, respectively, with a maximum of 1 sample per individual per time point.

### Untargeted Volatile Metabolome Analysis by GC-TOF-MS

There were no volatile metabolites discriminative for LOM compared to controls (false discovery rate > 0.1) in the 3 days prior to LOM diagnosis. Supplementary Table 9 shows the uncorrected *P* values of the Student *t* test for the 11 most significant metabolites. There was no overlap of significant metabolites between time points as analyzed by *t* test. In addition, class prediction by RF, which aimed to identify discriminative metabolite patterns, did distinguish LOM infants versus controls 2 days prior to diagnosis, with an AUC of 0.82 (95% CI, .64–.96) (Supplementary Table 10).

## DISCUSSION

We aimed to identify the preclinical fecal microbiota composition and volatile metabolome in preterm infants developing LOM, compared to matched controls. Employing 2 statistical methods, we discovered 6 overlapping microbiota features that contributed to the discrimination of LOM samples versus matched controls at various TIs. Additionally, an RF classification model accurately discriminated fecal LOM samples from control samples (AUC, 0.88 at 1–3 days pre-LOM). Fecal volatile metabolome patterns moderately discriminated LOM from controls (AUC, 0.74 at 1 day pre-LOM).

Limited data connected LOM to the gut. We demonstrated the association between an altered gut microbiota colonization and development of LOM. Consistent with our findings, an immature intestinal microbiota was recently associated with group B streptococcal meningitis in mice [22]. Furthermore, alterations in gut microbiota composition and the fecal volatile metabolome precede other acute diseases of preterm infants, including NEC and LOS [10–18, 30]. At the genus level, a decrease in *Bacteroides* and increase in *Escherichia/Shigella* contributed to the RF prediction model of LOM in this study. In NEC and LOS, enrichment of *Escherichia/Shigella* and decrease in relative abundance of *Bacteroides* have been observed [16, 17]. Both an increase in *Escherichia/Shigella* and decrease in *Bacteroides* have been implicated in the promotion of inflammation [31, 32], potentially heightening susceptibility for bacterial translocation.

Alterations in preterm infant microbiota associate with measurable fecal metabolic changes [33]. While no fecal markers have been studied for LOM, previous research identified differing CSF factors in comparison to controls. In a 19 versus 19 case-control study, untargeted combined liquid chromatography and GC with tandem MS demonstrated a high discriminative value for LOM in CSF (AUC, 0.94) [34]. Bacterial LOM exhibited perturbations in alanine, aspartate, and glutamate metabolism [34]. Another study identified CSF β-2-microglobuline (AUC, 0.93) as a discriminative biomarker for CNS inflammation [35]. Studies on CSF profiles have the advantage of analyzing meningitis closer to the infection site, namely in the CSF. Despite invasive lumbar puncture being the gold standard for LOM diagnosis, it takes 48–72 hours for definite results. Therefore, a method that is faster and preferably also noninvasive, and that indicates LOM at an early stage, may help in guiding swift antibiotic therapy directed at LOM.

Our study explored the potential of noninvasive methods complementing traditional diagnostic approaches. Indeed, with pattern recognition analysis (GC-IMS), volatile metabolome patterns from cases and controls could be discriminated with moderate accuracy (AUC, 0.74) at 1–3 days pre-LOM. However, specific volatile metabolites differentiating cases from controls were not identified with GC-TOF-MS. Consequently, we did not identify specific metabolites that could eventually lead to the development of a targeted VOC analytical device.

This study has several strengths, including its longitudinal design and standardized methodology regarding collection of clinical data and storage and handling of the fecal samples. The prospective, multicenter design of the study, the homogenous study population, and the relatively large initial sample size considering the low incidence rates of LOM were additional strengths. Sampling of all preterm infants admitted to the neonatal intensive care units enabled us to investigate microbial and metabolomics fingerprints of LOM before diagnosis. Furthermore, we utilized 2 distinct statistical methods for the analysis of the microbiota data, showing robustness and reproducibility of the results with different statistical analytical techniques, strengthening our conclusion of a differential preclinical LOM microbiota fingerprint.

However, this study is limited by several factors. Given the rarity of LOM, sample size was relatively small. Implementing various analytical techniques (fecal volatile metabolome vs microbiota) lowered available fecal sample mass per technique, necessitating rematching of microbiota cases to new controls, thereby removing the option to analyze the samples in a multiomics approach. In addition, despite the widespread use of 16S rRNA sequencing for the analysis of fecal microbiota composition, it lacks depth to classify bacteria at species or sometimes even genus level. Although the top abundant OTUs matched the causative agent in 6 of 8 infants (75%) with a positive CSF, it was impossible to investigate whether the specific causative LOM pathogen was abundant in the fecal microbiota before onset of disease.

Moreover, matching procedures did not include mode of delivery, which is known to influence gut microbiota composition in term infants; however, this influence is not evident in preterm infants [36]. In addition, delivery mode does not influence the composition of fecal VOCs in the headspace [37]. Therefore, we do not expect that delivery mode significantly impacted results. Furthermore, LOM was accompanied by LOS in 90% of the infants, making it impossible to exclude the effects of LOS on fecal patterns. Therefore, future studies should compare infants with LOM and LOS combined to infants with only LOS. In addition, the low number of cases made it impossible to perform separate subanalyses for infants with different causative pathogens of LOM. In LOS, the accuracy of fecal microbiota and volatile metabolome analysis has been shown to increase when specific pathogens are tested separately, which may also apply for LOM [10, 13, 14, 17]. Nevertheless, despite the heterogeneity of pathogens in both blood culture and liquor culture—including *Staphylococcus epidermidis*—predictive accuracy was higher than a previous LOS prediction model in preterm infants [13].

In 12 of the 21 LOM cases, no pathogen was cultured in the CSF. As liquor puncture is often delayed to postacute setting, after administration of antibiotics, the CSF may already be sterilized, resulting in false-negative CSF cultures. In the current study, 14 of 21 infants received antibiotics prior to lumbar puncture (Supplementary Table 1). The lack of a consensus on the definition of neonatal LOM in preterm infants, for example, clinical LOM versus LOM based on CSF culturing, additionally limits reproducible research between research groups [3]. Nonetheless, we meticulously included LOM cases based on predefined clinical and biochemical criteria.

Findings from this first explorative study require further investigation. Bacterial features identified through RF and MaAsLin2 require confirmation in an adequately powered study. In addition, the correlation between LOM and LOS has to be investigated, to determine whether LOM is a separate entity detectable with alterations in the gut microbiota and associated fecal volatile metabolome preceding diagnosis, or is rather a reflection of a preinflammatory state, similar to LOS and NEC. Utilizing novel in vitro techniques like 3-dimensional organoids and 2-dimensional monolayers to study epithelial, endothelial, and blood-brain barrier function and their interactions with LOM-specific gut bacteria, can enhance understanding of LOM pathophysiology [38]. Metagenomic sequencing could provide insight into the specific species and strains present before LOM, answering whether LOM-causing pathogens are present in the fecal microbiota before diagnosis. Establishing a causal role of disturbed gut microbiota in LOM pathogenesis or other acute neonatal diseases may be the foundation for developing a neonatal prediction model based on preclinical gut microbiota changes. Targeted modulation of the gut microbiota through probiotics, synbiotics, or antibiotics may prevent or alter disease course and mitigate the risk of neonatal diseases related to adverse outcomes.

To conclude, this is the first study reporting significant differences in the fecal microbiota and volatile metabolome between LOM and controls before clinical onset. This suggests that LOM, similarly to LOS, is preceded by a disturbed microbial colonization, and that bacteria causing LOM potentially originate from the gut. This could provide opportunities for development of novel diagnostic, therapeutic, and even preventive strategies for LOM.

## Supplementary Material

jiae265_Supplementary_Data

## Supplementary Data

Supplementary materials are available at *The Journal of Infectious Diseases* online (http://jid.oxfordjournals.org/). Supplementary materials consist of data provided by the author that are published to benefit the reader. The posted materials are not copyedited. The contents of all supplementary data are the sole responsibility of the authors. Questions or messages regarding errors should be addressed to the author.

## Notes

***Author contributions.*** N. F. and N. D. designed the study, collected data, drafted the initial manuscript, and revised the manuscript. N. F. analyzed the samples. S. M. H. designed the study, collected data, and critically reviewed the manuscript. A. A. was involved in statistical and machine learning analytics, drafted the initial manuscript, and revised the manuscript. M. N. Q. was involved in analysis of the samples, processing of microbiota data, and critically reviewed the manuscript for important intellectual content. E. D. was involved in sample analysis. A. W. and J. C. performed statistical and machine learning analytics and initial drafting of the paper, and critically reviewed the manuscript for important intellectual content. W. d. B., V. C., C. v. H., A. v. K., B. K., E. d. H., W. d. J., D. V., and M. v. W. collected data and critically reviewed the manuscript for important intellectual content. M. B., N. d. B., and J. v. G. conceptualized the study and critically reviewed the manuscript for important intellectual content. T. d. M. and H. N. conceptualized and designed the study and data collection, collected data, supervised data collection, and critically reviewed the manuscript for important intellectual content. All authors approved the final manuscript as submitted and agree to be accountable for all aspects of the work.

***Disclaimer***. The funding sources had no role in the design and conduct of the study.

***Funding*.** This work was supported by an unrestricted grant from the European Union Horizon 2020 Research and Innovation Program under the Marie Skłodowska-Curie grant agreement (grant number 814168); and unrestricted grant from the Stichting Zeldzame Ziekten Fonds.

***Data availability.*** The data are available through Figshare, doi: https://10.6084/m9.figshare.25370752.
