# Supplementary material for: Fecal Microbiota and Volatile Metabolome Pattern Alterations Precede Late-Onset Meningitis in Preterm Neonates
Source: J Infect Dis. 2024 May 23;231(6):1382–91. doi: 10.1093/infdis/jiae265 (PMC12247813; doi:10.1093/infdis/jiae265)
Supplement: jiae265_Supplementary_Data [file jiae265_supplementary_data.docx]

# Supplementary Material

1. **Gas chromatography – ion mobility spectrometry analysis**

The Gas chromatography – ion mobility spectrometry analysis (GC-IMS, FlavourSpec®, G.A.S., Dortmund, Germany) was fitted with a CTC PAL autosampler (CTC, Zwingen, Switzerland). The samples were analyzed as described by Rouvroye et al. [1]. The fecal samples were placed in 20ml glass vials with headspace caps and heated/agitated for eight minutes at 80˚C to generate sufficient headspace concentration. The molecules in the headspace were then injected into the GC-IMS instrument where they were first pre-separated by retention time based on chemical interactions with the GC column. They then enter the ion mobility spectrometer, where molecules were ionized by a low-radiation tritium (H3) source, creating reactant ions. Subsequently, these ions were moved by an electric field at atmospheric pressure against the flow of an inert drift gas (in this case nitrogen), creating nitrogen/ion collisions. These collisions result in the ions selectively losing momentum, extending the travel time through the drift-tube before being detected. Thus, the ions are separated based on a combination of their mass, charge and size. The ion current is detected by an electrometer as a function of time. For performed experiments, the selected conditions were as follows: the GC used a 15m, SE-54 column (CS Chromatographie, Germany) and was performed at 40˚C using nitrogen 99.9% (3.5 bar) as the carrier gas. The IMS used nitrogen as the drift gas and was performed at 45˚C. The flow rate for the nitrogen was 20 mL/min (34.175 kPa) for six min (GC), and 150 mL/min (0.364 kPa) (IMS).

1. **Gas chromatography – time of flight – mass spectrometry analysis**

Gas chromatography – time of flight – mass spectrometry (GC-TOF-MS) analysis was undertaken using a Markes Bench TOF-TD, connected to a Thermo Fisher GC fitted with a Rxi-624Sil column (length 20m, internal diameter 0.18mm, thickness 1.0 µm; Thames Restek, USA). Sample introduction was achieved using a Markes Unity Autosampler. The headspace was first concentrated from the vials onto bio-monitoring sorbent tubes from Markes (C2-AAXX-5149). This was done by heating the samples for 20 minutes at 40˚C before pumping the headspace onto the tubes for 20 minutes (5ml/minute) using a SKC Pocket Pump (SKC Ltd., Dorset, UK). The sorbent tubes were then loaded into the auto-sampler. The Markes TOF-DS software was used to add the unique identifier codes to the tubes and select the appropriate run sequence.

Subsequently, the headspace was pre-separated by GC before being injected into the TOF-MS transfer line. The molecules were ionized and accelerated by an electric field, resulting in the same kinetic energy for ions with a similar charge. The velocity of the ions is also determined by their mass (heavier ions with the same charge move slower). The time to reach the detector at a set distance was measured.

The GC calibration was as follows: the stand-by split of 150˚C was used with an overlap (to reduce overall run time), with the GC temperature ramping from 40˚C to 280˚C by 20˚C per minute with a total run time of 25 minutes. The desorption was performed by pre-purging the sample for one minute and then heating it for 10 minutes at 250˚C with a trap purge time of one minute. Subsequently, the trap was cooled to 30˚C and then purged for three minutes at 300˚C. Other settings were as follows: the filament voltage was set to 1.7 V (10s filament delay), transfer line was set to 250˚C, ion source temperature was 250˚C and electron ionization was performed at -70V. Masses from 35 to 350 atomic mass units were analyzed.

1. **Statistical analysis**

16S rRNA sequencing data

Phyla abundancies were compared between cases and controls by Wilcoxon Rank test. Diversity was analyzed using Richness, Chao1, and Shannon indices using ANOVA.

- 1. *Preprocessing of data*

All the microbiota data sets were normalized using auto scaling (mean-centered and divided by standard deviation (SD) of each variable). Data normalization was performed to compare the microbes in the same scale. We have considered 76 microbiota features in this analysis. We used multiple methods. First, unsupervised principal coordinate analysis (PCoA) was performed to investigate variations in the cohort between control versus LOM infants. Furthermore, Permutational Multivariate Analysis of Variance (PERMANOVA) was used to check the statistical significance of the clusters produced by the PCoA. In addition to the above methods, we used the supervised machine learning method Random Forest (RF) and Microbiome Multivariable Associations with Linear Models (MaAsLin 2) [2, 3].

- 1. *Random Forest analysis*

Random Forest (RF) machine learning ensemble method was applied to obtain optimal predicting performance out of the data, we employed [4]. This made it possible to analyze all the microbiome features in a nonlinear manner rather than just linearly, leading to the identification of more intricate connections between the microbiome features. For building a model (referred to as a training set) or for evaluating the performance of the model, RF employs bootstrapping techniques. With replacement, the bootstrapping procedure creates random samples from the dataset. Each bootstrapped sample has an associated “out-of-bag” (OOB) sample that is left out and is used to assess the algorithm’s effectiveness. In addition, we have used 10-fold cross validation to estimate the predictions. We have reported the OOB error rate in Table 3.

In order to categorize various response, i.e. class labels, we employed RF as a classification method. In case of the case vs. control samples, we used binary classification mode, whereas per time interval, we used RF as multi class classification mode. For instance, it was necessary to define the number of trees (ntree) and the number of variables (for instance, the number of genes) randomly picked as candidates at each split. In our models, we used ntree=500 and mtry=square root of the variables. To established the performance characteristics of the RF model, the area under curve (AUC) curve was calculated using only the OTUs that had an individual AUC>0.6. We have use R software (v 4.3.1) and MetaboAnalyst 6.0 software to perform Random Forest analysis [5].

- 1. *Multivariate association analysis with MaAsLin 2 (Microbiome Multivariable Associations with Linear Models 2.0)*

MaAsLin 2 is a R program designed specifically for performing multivariate association analysis of microbial meta-omics data [3]. Generalized linear models are utilized to cope with various study designs, including cross-sectional and longitudinal, by efficiently dealing with confounding factors and repeated measures. We used fixed effects as “DiseaseControl” OR “Timepoint” and random effects as “participant”. The R script can be found in the article of Mallick *et al.* (2021) [3]. A p-value<0.05 was considered significant. We selected the top MaAsLin2 features based on a p-value<0.1 for every time interval (Supplementary Tables S4-6).

- 1. *Principal Coordinates Analysis (PCoA)*

Principal Coordinates Analysis (PCoA), also known as Multidimensional Scaling (MDS), is a statistical technique used in the analysis of dissimilarity or distance matrices. PCoA extracts the main axes of variation also called principal components that explain the most variance in the distance matrix. In this analysis we have used Bray-Curtis distance matrix. The first few principal components (PCs) typically capture most of the variation in the data and hence we have used first two components.

- 1. Permutational Multivariate Analysis of Variance (PERMANOVA)

PERMANOVA, also known as Permutational Multivariate Analysis of Variance, is a robust statistical technique commonly used in microbiome research to evaluate variations in microbial community composition between different groups (For example: disease vs. control) . Finally, a permutation test is conducted, involving various rearrangements or randomized of group labels. The test statistic obtained from the data is compared to a distribution obtained by permuting the data, and if the observed statistic falls inside the extreme values of the distribution, it indicates substantial differences in microbial composition across the groups. The analysis result is visualized by PCoA analysis.

Volatile metabolome data

- 1. *Preprocessing of data*

As every analyzed sample generates a highly dimensional data set containing 11 million data points that are highly sparse with the majority of datapoints holding no useful information, the dimensionality of the GC-IMS-data was reduced before data processing was performed. This was done in two steps. First, as all the chemical information resides in the central portion of the data, we are able to crop the data without losing any chemical information. The crop parameters were identified by visual inspection and were referenced to the RIP (reactive ion peak). The same values were used for all the samples. Within the reduced dataset, a threshold was then applied to make all background datapoints zero. The value was selected to be twice the average of the background noise and the same value was applied to all the samples. This process reduces the number of non-zero data points to below 10,000, but without losing any chemical information.

The data rendered by GC-TOF-MS-analysis were processed differently. During the analysis of the samples, the TOF-DS™ software applied dynamic background compensation which automatically removed chromatogram background interference. This software also integrates and deconvolutes the peaks in the chromatogram. Subsequently, the present compounds were identified. Integration settings were as follows: Global Height Reject: 10000, Global Width Reject: 0.001, Baseline Threshold: 3, and Global Area Reject: 10000. The NIST (National Institute of Standards and Technology, 2022) database was used for identification of specific compounds. Both forward and reverse were matched with a minimum match factor of 450. Variables which were zero in ≥25% of samples, were excluded from further analysis, as these are unlikely to be of use for modelling. Additionally, potential contaminants were manually excluded. Argon, carbon dioxide and organosilanoles were removed from the dataset, as they might be derived from the column lining. In total, 170 unique metabolites remained. Once completed, all the data was combined and a simple t-test was used to identify relevant chemicals. This was done due to the small number of samples processed by GC-TOF-MS.

- 1. *Class prediction with machine learning of volatile metabolome data*

The data were analyzed as previously described [6-9]. A data analysis pipeline was developed in ‘R’ (version 4.0.3). Once the data had been pre-processed, it was then analyzed using a 10-fold cross validation approach. In this case, the data is divided into 10 equal groups with 9 groups being used as training data for the model and then the 10th group as the test set. This was repeated 10 times until all the samples had been a test sample. Within each fold a Wilcoxon rank-sum test was used to calculate the p-values for identification of the 20, 50, and 100 (GC-IMS) and 15 (GC-TOF-MS) most discriminatory features within the training set. The most discriminatory features were then used in a Random Forest model. This was applied to the test set, producing a probability for each sample. Once all the samples had been a test sample, the probabilities were collated and from its statistical performance parameters calculated, such as area under curve, sensitivity, and specificity.

# Supplementary Figures and Tables


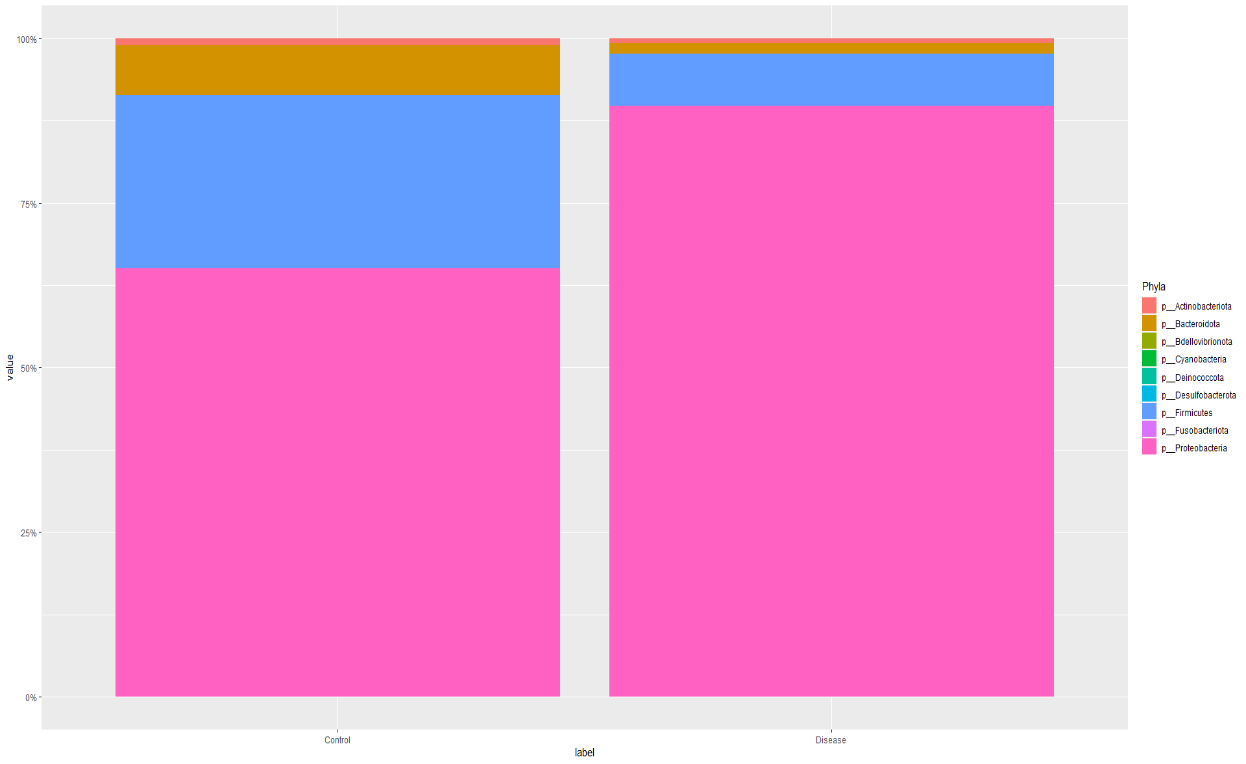


**Supplementary Figure S1**. Bacterial phyla in pooled samples from cases vs. controls. Mean relative abundances of taxa at phylum levels from pooled fecal samples 1 to 10 days pre-LOM (right bar) and matched postnatal age in controls (left bar). The relative abundance of the phyla of infants with LOM showed a trend towards an increased abundance of Proteobacteria, and decreased abundance of Firmicutes and Bacteriodetes.


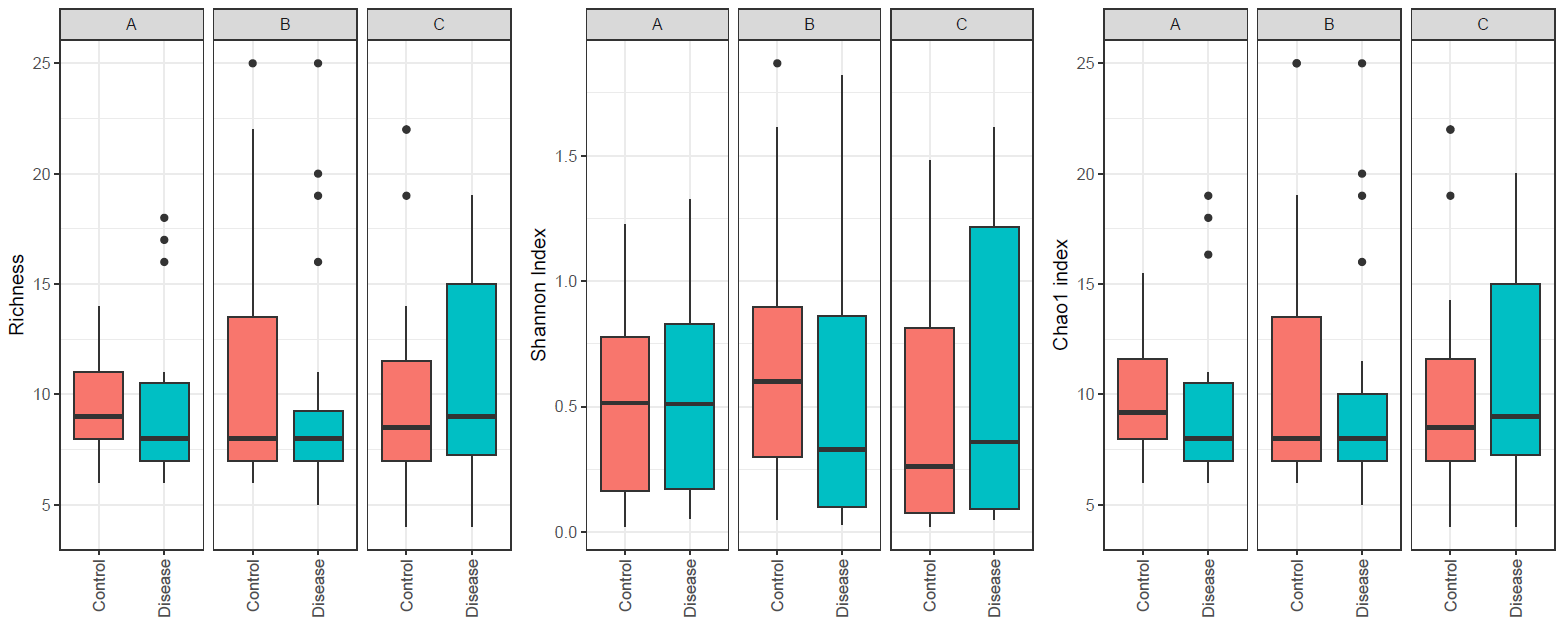


• Controls

• Cases (infants diagnosed with late-onset meningitis)

**Supplementary Figure S2.** Diversity analysis comparing samples from LOM infants versus samples from control infants at the different time intervals (A to C). No significant differences in diversity is found in species richness (Richness and Chao1 index) or species evenness (Shannon index).


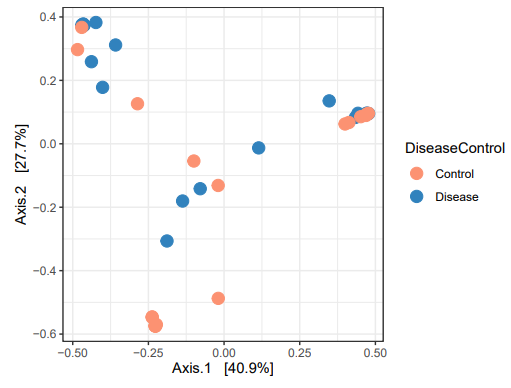

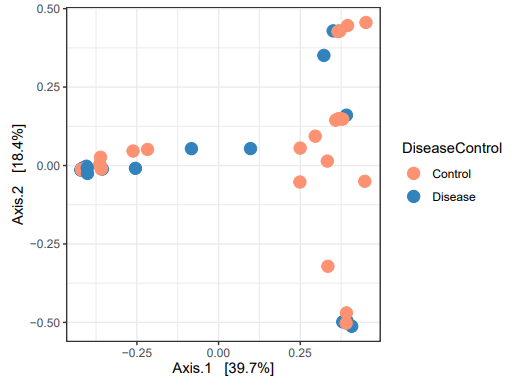


**C)**


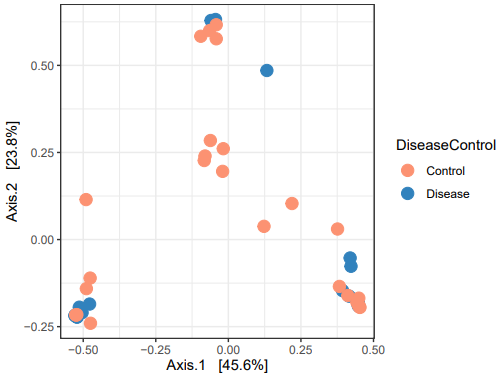

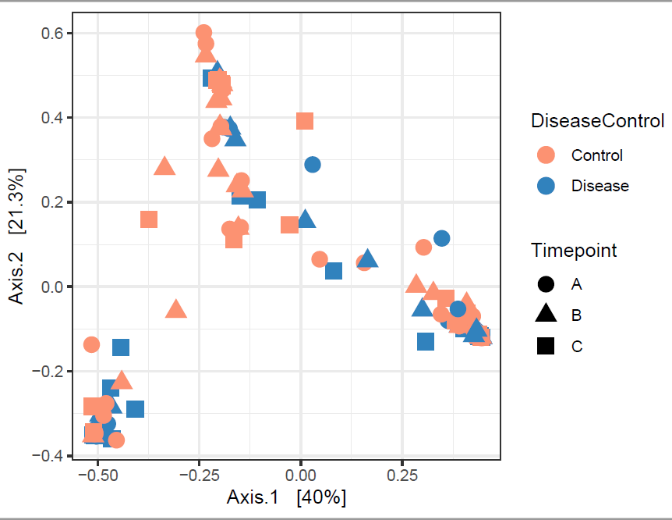


**A)**

**B)**

**D)**

**Supplementary Figure S3.** Principal Coordinates Analysis (PCoA) score plot performed using Bray-Curtis dissimilarity distance demonstrating variations across samples and time points. Orange and blue circles represent samples (disease or controls). Axis 1 and Axis 2 represents first and second principal components respectively. A) One to three days (time interval A), B) four to six days (time interval B), and C) seven to ten days (time interval C) (p-value<0.022) before onset of late-onset meningitis. D) All time intervals pooled together (p-value=0.002).


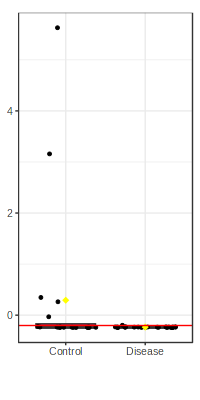

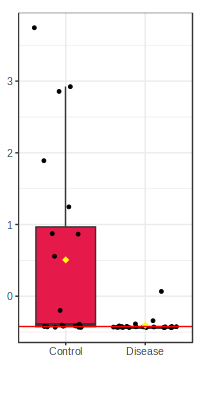

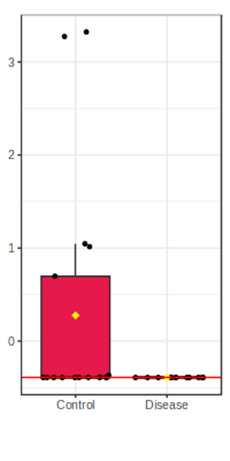

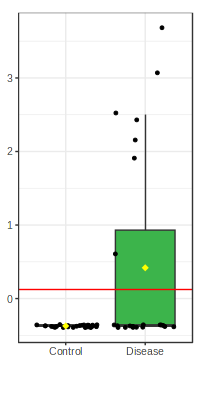


**D)**

**C)**

**A)**

**B)**

**Supplementary Figure S4.** The up- and downregulation of the bacterial features that are found as important for the discrimination of fecal late/onset meningitis samples versus control samples. The mean values of the microbiome are presented as yellow diamonds. The horizontal red lines indicate the optimized cut-off values of the microbiome expression. A) *Bacteroides* genus B) *Leuconostoc mesenteroides* species C) *Staphylococcos* genus D) *Corynebacterium* genus.


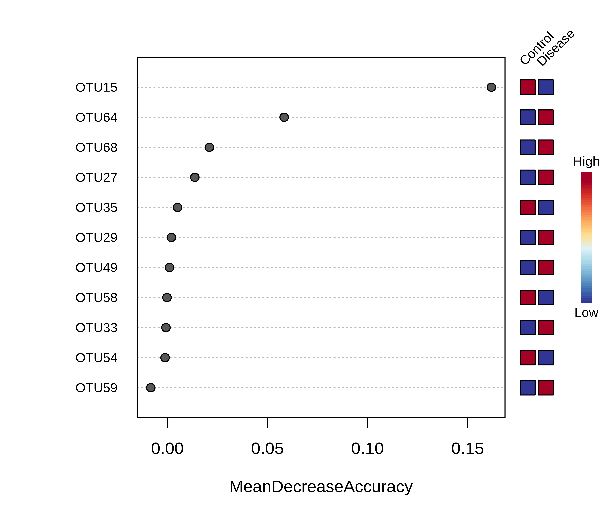

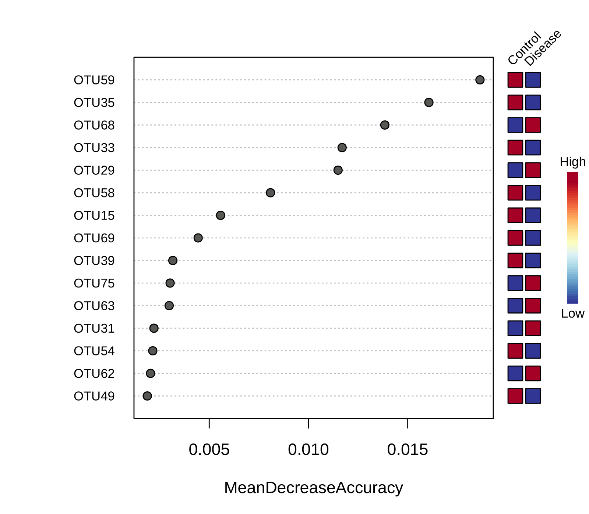

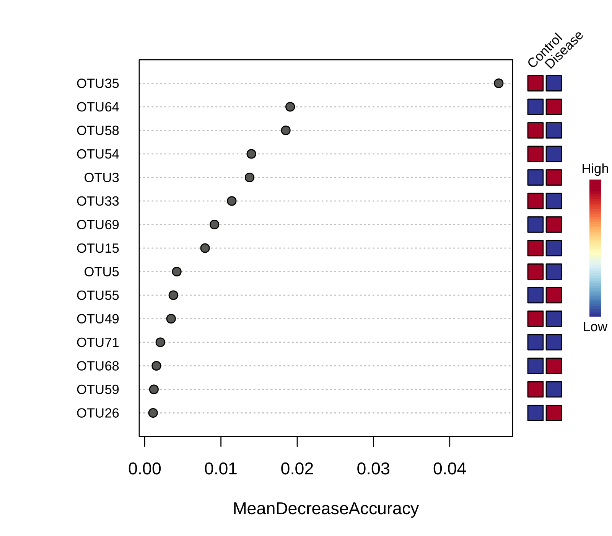


**C)**

**B)**

**A)**

**Supplementary Figure S5.** Using a Random Forest classification model, the variable importance was measured and the top operating taxonomic units (OTU) were ranked in terms of mean decrease in accuracy. Up/down regulation of the OTU in late/onset meningitis (LOM) cases is shown as a colored (red/blue) slide bar. One to three days (A), four to six days (B) and seven to ten days (C) before onset of late-onset meningitis are depicted. Supplementary Table S3 shows which bacterial features are connected to the OTUs.

| **Supplementary Table S1.** Detailed case-by-case description | | | | | | | | | | | | | | | | | | |
| --- | --- | --- | --- | --- | --- | --- | --- | --- | --- | --- | --- | --- | --- | --- | --- | --- | --- | --- |
| Centre | Postnatal age LOM (days) | Pathogen CSF culture | Culture confirmed LOS | Postnatal age LOS (days) | Pathogen blood culture | VP-drain in situ prior to LOM T0 | IVH grade prior to LOM T0 | Total antibiotic days prior to LOM T0 | AB <48 hours before lumbar puncture | Pleiocytosis in liquor (cells/µL) | Erytrocytes liquor (cells/µL) | Traumatic lumbar punction | CRP (mg/L) at LOM T0 | Duration LOM therapy (days) | Deceased | n GC-IMS | n GC-TOF-MS | n 16S rRNA analysis |
| 1 | 5 | No pathogen cultured | Yes | 5 | *Escherichia coli, Enterococcus faecalis* | No | NA | 3 | No | 51 | 3600 | No | 98.8 | 21 | No | 1 | 1 |  |
| 1 | 20 | *Moraxella osloensis* | Yes | 20 | *Serratia liquefaciens* | No | 2 | 7 | No | 24 | 3900 | Yes | 155.3 | 21 | No | 2 | 2 | 8 |
| 1 | 25 | *Group B Streptococcus* | Yes | 25 | *Group B Streptococcus* | No | NA | 3 | No | 15 | <900 | Yes | 23 | 2 | Yes | 3 | 3 | 8 |
| 1 | 14 | No pathogen cultured | Yes | 14 | *Serratia marcescens* | No | NA | 6 | No | 111 | <900 | No | 81.6 | 21 | No | 2 | 2 | 3 |
| 1 | 19 | *Klebsiella pneumoniae* | Yes | 18 | *Klebsiella pneumoniae* | No | NA | 4 | Yes | 35 | <300 | X | 102.9 | 20 | No | 1 |  |  |
| 1 | 7 | *Escherichia coli* | Yes | 7 | *Escherichia coli* | No | NA | 3 | No | 96 | <300 | No | 102.7 | 21 | No | 1 |  | 2 |
| 2 | 8 | No pathogen cultured | Yes | 5 | *Escherichia coli* | No | 2 | 6 | Yes | 542 | 232000 | Yes | 4 | 21 | No | 1 | 1 |  |
| 3 | 17 | *Serratia marcescens* | Yes | 16 | *Serratia marcescens* | No | NA | 4 | Yes | 100 | 96000 | Yes | 156 | 22 | No | 2 | 2 | 4 |
| 3 | 16 | *Escherichia coli* | Yes | 17 | *Escherichia coli* | No | 2 | 6 | Yes | 393 | 35441 | Yes | 64 | 7 | Yes |  |  | 3 |
| 3 | 10 | No pathogen cultured | Yes | 10 | *Enterobacter cloacae* | No | 2 | 3 | Yes | 1100 | 1 | No | 63 | 7 | Yes | 3 | 3 | 5 |
| 4 | 20 | *Escherichia coli* | No |  | No pathogen cultured | No | 2 | 8 | Yes | 3820 | 4000 | Yes | 60 | 21 | No | 2 | 2 | 5 |
| 6 | 10 | *Enterobacter cloacae* | Yes | 9 | *Enterobacter cloacae* | No | 1 | 1 | Yes | 856 | X | No | 73.5 | 21 | No | 1 | 3 | 5 |
| 6 | 22 | No pathogen cultured | Yes | 7, 22 | 7: *Enterococcus faecalis*, 22: *Klebsiella oxytoca* | No | NA | 8 | No | 6332 | 1 | No | 2.7 | 28 | No | 3 | 2 | 5 |
| 7 | 8 | No pathogen cultured | Yes | 7 | *Staphylococcus epidermidis* | No | NA | 3 | Yes | 35 | 200 | No | 92 | 14 | No | 3 | 1 |  |
| 7 | 14 | No pathogen cultured | Yes | 13 | *Escherichia coli* | No | 1 | 2 | Yes | 166 | 1200 | Yes | 89 | 21 | No | 2 | 2 | 7 |
| 7 | 13 | *Staphylococcus epidermidis* | Yes | 11 | *Escherichia coli* | No | 1 | 2 | Yes | 248 | 700 | Yes | 114 | 21 | No | 2 | 2 | 6 |
| 7 | 7 | No pathogen cultured | Yes | 7 | *Staphylococcus epidermidis* | No | 1 | 3 | Yes | 195 | 7300 | Yes | 75 | 14 | No | 1 |  |  |
| 8 | 9 | No pathogen cultured | No |  | No pathogen cultured | No | NA | 9 | Yes | 75 | 4523 | Yes | 45 | 21 | No | 3 | 3 | 5 |
| 8 | 10 | No pathogen cultured | Yes | 9, 11 | 9: Staphylococcus epidermidis 11: Enterobacter cloacae | No | NA | 7 | Yes | 278 | 23000 | Yes | 85 | 21 | No | 2 |  |  |
| 8 | 19 | No pathogen cultured | Yes | 19 | Staphylococcus epidermidis | No | NA | 9 | No | 74 | 217 | No | 116 | 14 | No | 3 | 2 |  |
| 8 | 12 | No pathogen cultured | Yes | 12 | Klebsiella variicola | No | NA | 3 | Yes | 3830 | 605 | No | 24 | 21 | Yes | 3 | 1 | 3 |
| Median  (IQR) | | | | | | | | | | | | | | | | 2 (2) | 2 (1) | 5 (3) |
| Abbreviations: LOM, late-onset meningitis; LOS, late-onset sepsis; CSF, cerebrospinal fluid; T0, postnatal age of onset; VP, ventriculoperitoneal; IVH, intraventricular hemorrhage; AB, antibiotics; NA, not applicable; GC-IMS, gas chromatography – ion mobility spectrometry; GC-TOF-MS, gas chromatography – time of flight – mass spectrometry IQR, interquartile range; X, unknown; n, number of samples. | | | | | | | | | | | | | | | | | | |

| Supplementary Table S2. Operating taxonomic units (OTUs) that are selected by the Random Forest classification model as important for the discrimination of late-onset meningitis samples versus control samples. | |
| --- | --- |
| OTU3 | *Bifidobacterium* genus |
| OTU5 | *Corynebacterium* genus |
| OTU15 | *Bacteroides* genus |
| OTU26 | *Geobacillus* genus |
| OTU27 | *Enterococcus* genus |
| OTU29 | *Leuconostoc mesenteroides* species |
| OTU31 | *Streptococcus* genus |
| OTU33 | *Streptococcus salivarius* species |
| OTU35 | *Staphylococcus* genus |
| OTU39 | *Clostridium perfringens* species |
| OTU49 | *Veillonella* genus |
| OTU54 | *Afipia_uncultured bacterium* species |
| OTU55 | *Bradyrhizobium* genus |
| OTU58 | *Ralstonia* genus |
| OTU59 | *Comamondaceae* family |
| OTU62 | Enterobacteriales order |
| OTU63 | *Enterobacteriaceae* family |
| OTU64 | *Escherichia-Shigella* genus |
| OTU68 | *Yersinaceae* family |
| OTU69 | *Serratia* genus |
| OTU71 | *Pasteurelaceae* family |
| OTU75 | *Pseudomonas* genus |

| **Supplementary Table S3**. Microbiota features with a p-value<0.1 as selected by MaAsLin 2 at the three different time intervals including coefficients and standard error. | | | | |
| --- | --- | --- | --- | --- |
| **Time interval** | **Microbiota feature** | **Coefficients** | **Standard Error** | **p-value** |
| TI-A | *Bacteroides* genus | -2.909 | 1.217 | **0.024*** |
|  | *Yersiniaceae* family | 1.620 | 0.762 | **0.044*** |
| TI-B | *Yersiniaceae* family | 1.646 | 0.810 | 0.054 |
|  | *Leuconostoc mesenteroides* species | 0.503 | 0.265 | 0.071 |
| TI-C | *Corynebacterium* genus | -1.326 | 0.548 | **0.020*** |
|  | *Staphylococcus* genus | -2.531 | 1.284 | 0.062 |
|  | *Bifidobacterium* family | 0.570 | 0.315 | 0.086 |
| *TI, time interval; MaAsLin2, Microbiome Multivariable Associations with Linear Models 2.0*  **p-value<0.05 considered significant* | | | | |

| **Supplementary Table S4**. Microbiota features selected by MaAsLin 2 at time interval A, including coefficients, standard error, q-values, and p-values. | | | | |
| --- | --- | --- | --- | --- |
| **Microbe features** | **Coefficients** | **Standard Error** | **p-value** | **q-value** |
| *Bacteroides* genus | -2,909 | 1,217 | **0,024*** | 0,394 |
| *Yersiniaceae* family | 1,620 | 0,762 | **0,044*** | 0,394 |
| *Enterobacterales* order | -1,096 | 0,829 | 0,198 | 0,918 |
| *Staphylococcus* genus | -1,097 | 1,016 | 0,291 | 0,918 |
| *Escherichia-Shigella* genus | 1,929 | 1,880 | 0,315 | 0,918 |
| *Streptococcus* genus | 0,674 | 0,780 | 0,395 | 0,918 |
| *Afipia* genus; uncultered_bacterium | 0,271 | 0,307 | 0,395 | 0,918 |
| *Clostridium_perfringens* species | -0,768 | 0,913 | 0,408 | 0,918 |
| *Bifidobacterium* genus | 0,877 | 1,396 | 0,535 | 0,970 |
| *Bacteroides_vulgatus* species | 0,530 | 0,955 | 0,584 | 0,970 |
| *Enterobacteriaceae* family | 0,758 | 1,400 | 0,593 | 0,970 |
| *Veillonella* genus | -0,576 | 1,949 | 0,770 | 0,974 |
| *Finegoldia* genus; uncultered_bacterium | 0,209 | 0,708 | 0,771 | 0,974 |
| *Ralstonia* genus | -0,169 | 0,654 | 0,799 | 0,974 |
| *Serratia* genus | -0,194 | 1,271 | 0,880 | 0,974 |
| *Streptococcus_salivarius* species | 0,044 | 0,409 | 0,915 | 0,974 |
| *Comamonadaceae* family | 0,033 | 0,631 | 0,959 | 0,974 |
| *Enterococcus* genus | 0,046 | 1,417 | 0,974 | 0,974 |
| *Time interval A includes samples from one to three days before clinical onset of late-onset meningitis.*  *MaAsLin2, Microbiome Multivariable Associations with Linear Models 2.0*  **p-value<0.05 considered significant* | | | | |

| **Supplementary Table S5**. Microbiota features selected by MaAsLin 2 at time interval B, including coefficients, standard error, q-values, and p-values. | | | | |
| --- | --- | --- | --- | --- |
| **Microbe features** | **Coefficients** | **Standard Error** | **p-value** | **q-value** |
| *Yersiniaceae* family | 1,646 | 0,810 | 0,054 | 0,765 |
| *Leuconostoc_mesenteroides* species | 0,503 | 0,265 | 0,071 | 0,765 |
| *Streptococcus_salivarius* species | -0,855 | 0,569 | 0,147 | 0,765 |
| *Bacteroides* genus | -2,102 | 1,483 | 0,171 | 0,765 |
| *Streptococcus* genus | 1,217 | 0,949 | 0,213 | 0,765 |
| *Enterobacteriaceae* family | 1,640 | 1,552 | 0,302 | 0,765 |
| *Ralstonia* genus | -1,612 | 1,531 | 0,304 | 0,765 |
| *Corynebacterium* genus | -0,538 | 0,525 | 0,311 | 0,765 |
| *Comamonadaceae* family | -1,308 | 1,274 | 0,316 | 0,765 |
| *Staphylococcus* genus | -1,358 | 1,330 | 0,319 | 0,765 |
| *Haemophilus* genus | -0,450 | 0,530 | 0,406 | 0,885 |
| *Veillonella* genus | 1,262 | 1,687 | 0,464 | 0,889 |
| *Clostridium_perfringens* species | -0,618 | 1,155 | 0,597 | 0,889 |
| *Bradyrhizobium* genus | -0,162 | 0,362 | 0,659 | 0,889 |
| *Afipia* genus; uncultered_bacterium | -0,468 | 1,081 | 0,669 | 0,889 |
| *Bacteroides_vulgatus* species | -0,521 | 1,486 | 0,729 | 0,889 |
| *Escherichia-Shigella* genus | 0,540 | 1,544 | 0,730 | 0,889 |
| *Enterococcus* genus | 0,408 | 1,255 | 0,748 | 0,889 |
| *Bifidobacterium* genus | 0,389 | 1,202 | 0,749 | 0,889 |
| *Acinetobacter* genus | -0,161 | 0,518 | 0,759 | 0,889 |
| *Micrococcaceae* family | -0,103 | 0,400 | 0,800 | 0,889 |
| *Sphingomonas* genus | -0,144 | 0,608 | 0,815 | 0,889 |
| *Serratia* genus | 0,099 | 1,351 | 0,942 | 0,955 |
| *Enterobacterales* order | 0,056 | 0,978 | 0,955 | 0,955 |
| *Yersiniaceae* family | 1,646 | 0,810 | 0,054 | 0,765 |
| *Time interval B includes samples from four to six days before clinical onset of late-onset meningitis.*  *MaAsLin2, Microbiome Multivariable Associations with Linear Models 2.0* | | | | |

| **Supplementary Table S6**. Microbiota features selected by MaAsLin 2 at time interval B, including coefficients, standard error, q-values, and p-values. | | | | |
| --- | --- | --- | --- | --- |
| **Microbe features** | **Coefficients** | **Standard Error** | **p-value** | **q-value** |
| *Corynebacterium* genus | -1,326 | 0,548 | **0,020*** | 0,466 |
| *Staphylococcus* genus | -2,531 | 1,284 | 0,062 | 0,657 |
| *Bifidobacterium* genus | 0,570 | 0,315 | 0,086 | 0,657 |
| *Yersiniaceae* family | 1,201 | 0,901 | 0,196 | 0,680 |
| *Haemophilus* genus | -1,021 | 0,770 | 0,199 | 0,680 |
| *Geobacillus* genus | 0,467 | 0,352 | 0,207 | 0,680 |
| *Escherichia-Shigella* genus | 2,575 | 1,974 | 0,207 | 0,680 |
| *Micrococcaceae* family | -0,398 | 0,446 | 0,377 | 0,886 |
| *Enterococcus* genus | 0,854 | 1,049 | 0,425 | 0,886 |
| *Bradyrhizobium* genus | 0,410 | 0,501 | 0,426 | 0,886 |
| *Streptococcus* genus | 0,848 | 1,088 | 0,444 | 0,886 |
| *Enterobacterales* order | 0,854 | 1,197 | 0,485 | 0,886 |
| *Ralstonia* genus | -1,074 | 1,559 | 0,501 | 0,886 |
| *Acinetobacter* genus | -0,401 | 0,661 | 0,562 | 0,901 |
| *Comamonadaceae* family | -0,694 | 1,253 | 0,588 | 0,901 |
| *Bacteroides* genus | -0,810 | 1,753 | 0,649 | 0,905 |
| *Finegoldia* genus | 0,352 | 0,895 | 0,698 | 0,905 |
| *Enterobacteriaceae* family | 0,584 | 1,540 | 0,708 | 0,905 |
| *Bacteroides_vulgatus* species | 0,283 | 1,128 | 0,804 | 0,941 |
| *Serratia* genus | 0,285 | 1,221 | 0,818 | 0,941 |
| *Afipia* genus | -0,213 | 1,418 | 0,882 | 0,951 |
| *Sphingomonas* genus | 0,077 | 0,668 | 0,910 | 0,951 |
| *Veillonella* genus | -0,025 | 1,653 | 0,988 | 0,988 |
| *Time interval C includes samples from seven to ten days before clinical onset of late-onset meningitis.*  *MaAsLin2, Microbiome Multivariable Associations with Linear Models 2.0*  **p-value<0.05 considered significant* | | | | |

| **Supplementary Table S7.** The top 5 abundant OTUs in the microbiota of preterm infants with late-onset meningitis. In case of a positive CSF culture, the closest OTU found in our dataset that matched the causative agent in the CSF is shown. | | | | | | | | | | | | | | | | | | | | | | | | |
| --- | --- | --- | --- | --- | --- | --- | --- | --- | --- | --- | --- | --- | --- | --- | --- | --- | --- | --- | --- | --- | --- | --- | --- | --- |
| **Meningitis pathogen in CSF culture** | **Closest OTU to meningitis pathogen** | **Closest OTU in top 5 abundance** | **g__Bifidobacterium** | **g__Bacteroides** | **s__Bacteroides_vulgatus** | **g__Enterococcus** | **s__Leuconostoc_mesenteroides** | **g__Streptococcus** | **s__Streptococcus_salivarius** | **g__Staphylococcus** | **g__Clostridium_sensu_stricto_1** | **s__Clostridium_perfringens** | **g__Veillonella** | **g__Afipia;s__uncultured_bacterium** | **g__Ralstonia** | **f__Comamonadaceae** | **o__Enterobacterales** | **f__Enterobacteriaceae** | **g__Escherichia-Shigella** | **g__Pantoea** | **g__Hafnia-Obesumbacterium** | **f__Yersiniaceae** | **g__Serratia** | **g__Haemophilus** |
|  |  |  | **OTU3** | **OTU15** | **OTU16** | **OTU27** | **OTU29** | **OTU31** | **OTU33** | **OTU35** | **OTU38** | **OTU39** | **OTU49** | **OTU54** | **OTU58** | **OTU59** | **OTU62** | **OTU63** | **OTU64** | **OTU66** | **OTU67** | **OTU68** | **OTU69** | **OTU72** |
| *Moraxella osloensis* | No close OTU found | n.a. |  |  |  |  |  |  |  | X |  |  |  |  |  |  |  | X | X |  |  | X | X |  |
| *Group B Streptococcus* | OTU31 | Yes |  |  |  |  |  | O |  |  |  | X |  |  |  |  |  | X |  |  | X | X |  |  |
| No pathogen cultured | n.a. | n.a. |  |  |  |  |  |  |  |  |  |  | X |  |  |  |  | X |  | X |  |  | X | X |
| *Escherichia coli* | OTU64 | Yes | X |  |  |  |  |  |  | X |  |  |  |  |  |  |  | X | O |  |  |  | X |  |
| Serratia marcescens | OTU69 | Yes | X |  |  |  |  |  |  | X |  |  | X |  |  |  |  |  |  | X |  |  | O |  |
| *Escherichia coli* | OTU64 | No |  |  |  |  |  | X |  | X |  |  |  |  |  |  | X | X |  |  |  |  | X |  |
| No pathogen cultured | n.a. | n.a. |  |  |  | X |  |  |  | X |  |  | X |  |  |  |  | X | X |  |  |  |  |  |
| *Escherichia coli* | OTU64 | Yes |  |  |  | X |  |  |  | X |  |  |  |  |  |  |  | X | O |  |  |  | X |  |
| *Enterobacter cloacae* | OTU63 | Yes |  |  |  |  |  |  |  | X |  |  |  | X | X | X |  | O |  |  |  |  |  |  |
| No pathogen cultured | n.a. | n.a. |  |  |  | X |  |  |  |  | X |  |  |  |  |  | X | X |  |  |  |  | X |  |
| No pathogen cultured | n.a. | n.a. |  |  |  |  | X |  |  | X |  |  |  |  |  |  |  | X | X |  |  | X |  |  |
| *Staphylococcus epidermidis* | OTU35 | Yes | X |  | X |  |  |  |  | O |  |  |  |  |  |  |  | X | X |  |  |  |  |  |
| No pathogen cultured | n.a. | n.a. |  |  |  |  |  |  |  |  |  |  |  | X | X | X |  | X | X |  |  |  |  |  |
| No pathogen cultured | n.a. | n.a. |  | X |  |  |  |  |  | X |  |  |  |  |  | X |  | X | X |  |  |  |  |  |
| X, OTU present in top 5 abundance; O, OTU matches to the closest OTU of the causative meningitis pathogen.  n.a., not applicable; OTU, operating taxonomic unit; CSF, cerebrospinal fluid; g, genus; s, species; f, family. | | | | | | | | | | | | | | | | | | | | | | | | |

| **Supplementary Table S8.** Performance characteristics of Gas-chromatography – ion mobility spectrometry as analyzed by Random Forest with 50 and 100 features. | | | | | | | | | |
| --- | --- | --- | --- | --- | --- | --- | --- | --- | --- |
| **Features** | **Time point**  **(days)** | **Control samples (n)** | **Case samples (n)** | **P-value** | **AUC [95%CI]** | **Sensitivity [95%CI]** | **Specificity [95%CI]** | **PPV** | **NPV** |
| 50 features | t-1 | 15 | 18 | ***0.037*** | 0.68 [0.50-0.87] | 1.00 [0.78-1.00] | 0.67 [0.41-0.87] | 0.56 | 1.00 |
|  | t-2 | 13 | 19 | ***0.028*** | 0.70 [0.51-0.89] | 0.69 [0.39-0.91] | 1.00 [0.82-1.00] | 1.00 | 0.68 |
|  | t-3 | 13 | 14 | ***0.013*** | 0.75 [0.56-0.94] | 0.92 [0.64-1.00] | 0.57 [0.29-0.82] | 0.67 | 0.89 |
| 100 features | t-1 | 15 | 18 | 0.079 | 0.64 [0.45-0.64] | 1.00 [0.78-1.00] | 0.72 [0.47-0.90] | 0.54 | 1.00 |
|  | t-2 | 13 | 19 | ***0.018*** | 0.72 [0.53-0.72] | 0.62 [0.32-0.86] | 1.00 [0.82-1.00] | 1.00 | 0.70 |
|  | t-3 | 13 | 14 | ***0.024*** | 0.72 [0.52-0.72] | 0.92 [0.64-1.00] | 0.50 [0.23-0.77] | 0.63 | 0.88 |
| NPV: negative predictive value; PPV: positive predictive value; t-1. t-2. t-3: one day before. two days before and three days before clinical diagnosis of late-onset meningitis. resp., with one sample per individual per time point. | | | | | | | | | |

| Supplementary Table S9. Uncorrected unpaired t-test results for unequal variances of gas chromatography – time of flight – mass spectrometry (area under the curve of peak concentration) per metabolite of interest per time point. | | | |
| --- | --- | --- | --- |
| Time point | **t-3** | **t-2** | **t-1** |
| Number of samples (cases vs. controls) | **11 vs. 9** | **11 vs. 10** | **12 vs. 12** |
| 1-Propene. 2-methyl- | **0.04*** | 0.94 | / |
| 2H-1.2-Oxazine. 6-(4-chlorophenyl)tetrahydro-2-methyl- | **0.04*** | 0.34 | 0.97 |
| 3-Hydroxy-4-methoxybenzaldehyde. TBDMS | **0.03*** | 0.46 | 0.23 |
| Acetophenone | / | 0.08 | 0.78 |
| Butane. 2-methyl- | 0.31 | 0.42 | 0.36 |
| Carbon disulfide | 0.65 | 0.22 | 0.09 |
| Hexanal | 0.27 | 0.54 | 0.38 |
| Isopropyl Alcohol | 0.23 | 0.18 | 0.52 |
| Methylene chloride | 0.38 | 0.22 | 0.07 |
| Propanoic acid | 0.23 | 0.48 | / |
| Undecane | 0.17 | 0.49 | 0.44 |
| t-1, t-2, t-3: the day before, two days before and three days before clinical diagnosis of late-onset meningitis, respectively. *raw *P*-value <0.05 | | | |

| **Supplementary Table S10.** Performance characteristics of Gas-chromatography – time of flight – mass spectrometry as analyzed by Random Forest based on 15 features | | | | | | | | |  |
| --- | --- | --- | --- | --- | --- | --- | --- | --- | --- |
| **Time point**  **(days)** | **Control samples (n)** | **Case samples (n)** | ***P*-value** | **AUC [95%CI]** | **Sensitivity [95%CI]** | **Specificity [95%CI]** | **PPV** | **NPV** | |
| t-1 | 12 | 12 | 0.083 | 0.71 [0.50-0.90] | 0.83 [0.63-1.00] | 0.66 [0.44-0.90] | 0.80 | 0.63 | |
| t-2 | 10 | 11 | **0.014*** | 0.82 [0.64-0.96] | 0.70 [0.44-0.92] | 0.63 [0.37-0.87] | 0.70 | 0.71 | |
| t-3 | 9 | 11 | 0.425 | 0.61 [0.39-0.81] | 0.66 [0.40-0.90] | 0.64 [0.38-0.88] | 0.57 | 0.63 | |
| NPV: negative predictive value; PPV: positive predictive value; t-1, t-2, t-3: the day before, two days before and three days before clinical diagnosis of late-onset meningitis, respectively, with a maximum of one sample per individual per time point. **p-value<0.05 considered significant* | | | | | | | | |  |

**References**

1. Rouvroye, M.D., et al., *Faecal Scent as a Novel Non-Invasive Biomarker to Discriminate between Coeliac Disease and Refractory Coeliac Disease: A Proof of Principle Study.* Biosensors, 2019. **9**(2): p. 69.

2. Hu, J. and S. Szymczak, *A review on longitudinal data analysis with random forest.* Brief Bioinform, 2023. **24**(2).

3. Mallick, H., et al., *Multivariable association discovery in population-scale meta-omics studies.* PLoS Comput Biol, 2021. **17**(11): p. e1009442.

4. Acharjee, A., et al., *A random forest based biomarker discovery and power analysis framework for diagnostics research.* BMC Medical Genomics, 2020. **13**(1): p. 178.

5. Pang, Z., et al., *MetaboAnalyst 5.0: narrowing the gap between raw spectra and functional insights.* Nucleic Acids Res, 2021. **49**(W1): p. W388-W396.

6. Tiele, A., et al., *Breath Analysis Using eNose and Ion Mobility Technology to Diagnose Inflammatory Bowel Disease-A Pilot Study.* Biosensors (Basel), 2019. **9**(2).

7. Tiele, A., et al., *Breath-based non-invasive diagnosis of Alzheimer's disease: a pilot study.* J Breath Res, 2020. **14**(2): p. 026003.

8. Daulton, E., et al., *The Detection of Wound Infection by Ion Mobility Chemical Analysis.* Biosensors (Basel), 2020. **10**(3).

9. Lacey, L., et al., *Detection of Group B Streptococcus in pregnancy by vaginal volatile organic compound analysis: a prospective exploratory study.* Transl Res, 2020. **216**: p. 23-29.
